# Potential Residential Exposure to Toxics Release Inventory Chemicals during Pregnancy and Childhood Brain Cancer

**DOI:** 10.1289/ehp.9145

**Published:** 2006-06-06

**Authors:** Hannah S. Choi, Youn K. Shim, Wendy E. Kaye, P. Barry Ryan

**Affiliations:** 1 Department of Environmental and Occupational Health, Rollins School of Public Health, Emory University, Atlanta, Georgia, USA; 2 Division of Health Studies, Agency for Toxic Substances and Disease Registry, Atlanta, Georgia, USA

**Keywords:** air emissions, astrocytoma, brain cancer, children, GIS, PNET, pregnancy, Toxics Release Inventory

## Abstract

**Background:**

Although the susceptibility of the developing fetus to various chemical exposures is well documented, the role of environmental chemicals in childhood brain cancer etiology is not well understood.

**Objectives:**

We aimed to evaluate whether mothers of childhood brain cancer cases had greater potential residential exposure to Toxics Release Inventory (TRI) chemicals than control mothers during pregnancy.

**Methods:**

We included 382 brain cancer cases diagnosed at < 10 years of age from 1993 through 1997 who were identified from four statewide cancer registries. One-to-one matched controls were selected by random-digit dialing. Computer-assisted telephone interviews were conducted. Using residential history of mothers during pregnancy, we measured proximity to TRI facilities and exposure index, including mass and chemicals released. We calculated odds ratios (ORs) and 95% confidence intervals (CIs) using conditional logistic regression to estimate brain cancer risk associated with TRI chemicals.

**Results:**

Increased risk was observed for mothers living within 1 mi of a TRI facility (OR = 1.66; 95% CI, 1.11–2.48) and living within 1 mi of a facility releasing carcinogens (OR = 1.72; 95% CI, 1.05–2.82) for having children diagnosed with brain cancer before 5 years of age, compared to living > 1 mi from a facility. Taking into account the mass and toxicity of chemical releases, we found a nonsignificant increase in risk (OR = 1.25; 95% CI, 0.67–2.34) comparing those with the lowest versus highest exposure index.

**Conclusions:**

Risk of childhood brain cancers may be associated with living near a TRI facility; however, this is an exploratory study and further studies are needed.

There is significant concern about exposure of the fetus to environmental pollutants, food additives, and drugs, which may reach the fetus through the mother and affect the brain at critical stages of development. The developing central nervous system (CNS) is much more susceptible to chemical exposures than the adult CNS, and the brain is also the major target of toxicity for congenital effects. Some toxic agents impenetrable to the adult brain freely enter the developing brain because the blood–brain barrier of the fetus is not fully developed and is not completed until approximately 6 months after birth (Adinolfi 1985; Johanson 1980; Rodier 1994, 1995). Exposures to chemicals early in life are likely to have a greater impact on health outcomes such as cancer, neurodevelopmental impairment, and immune dysfunction (Thomas 1995).

Although the susceptibility of the developing fetus to various chemical exposures is well documented, the role of environmental chemicals in childhood brain cancer etiology is not well understood. The best established environmental risk factor for childhood brain cancer is radiation exposure (Harvey et al. 1985; Kuijten and Bunin 1993; MacMahon 1962; Mole 1974; Ron et al. 1988). Therapeutic cranial irradiation (X rays) has repeatedly been linked to childhood brain cancer, whereas diagnostic X rays with their usual low dose and short exposure periods were not enough to result in disease outcome (Kuijten and Bunin 1993).

Some studies investigated the risk for childhood cancer and birth defects among people living near hazardous waste sites and as a result of chemical exposures from the environment. Residential location has been a concern because toxic chemicals in landfill may disperse into the air or soil, eventually leading to human exposure (Elliot et al. 2001). In Clinton County, Pennsylvania, increased risk for cancer death was observed near the Drake Superfund site (Budnick et al. 1984). Other studies have attempted to find a link between childhood cancers and residential proximity to hazardous waste sites, with mixed results (Knox 2000; White and Aldrich 1999). In the Dover Township case–control study, a significantly increased odds ratio was observed for potential exposure to ambient air pollutants among female children with leukemia. However, no significant risks were found for brain cancer (New Jersey Department of Health and Senior Services and Agency for Toxic Substances and Disease Registry 2003).

One source of information on environmental contaminants is the Toxics Release Inventory (TRI), which is managed by the U.S. Environmental Protection Agency (U.S. EPA 2006). The TRI was established by a mandate of the Emergency Planning and Community Right-To-Know Act (EPCRA) of 1986. The TRI database contains an annual report of chemical releases to the environment and transfers of chemicals to off-site locations. The TRI captures the mass of specific compounds released into the environment (routinely or by accident) and those otherwise managed as waste. The mass of compounds released is considered to be relatively constant over the reporting period because what is released routinely and as waste usually exceeds what is accidentally released. As of 2002, > 650 toxic chemicals and chemical compounds are required to be reported.

The TRI database has been used in several studies. A link to slight increases in risk for certain birth defects associated with toxic releases (Marshall et al. 1997) has been suggested, but potential links to childhood cancers have not yet been investigated. The most common use of the TRI database has been to add up the total mass of TRI chemicals released to identify the most problematic polluters. However, this method emphasizes volume without regard to toxicity or environmental fate. Further, the total mass of chemicals released does not equal actual concentrations in the environment nor actual exposures to populations (Neumann 1998).

Previous studies used several different methods of categorizing exposure. Marshall et al. (1997) evaluated the risk of CNS and musculoskeletal birth defects from exposure to solvents, metals, and pesticides from hazardous contaminant sites including TRI sites in New York State. This case–control study rated the probability of exposure as “high,” “medium,” “low,” or “unknown” for each contaminant group, using a standard, 1-mi radius template divided into 25 sectors. The templates were centered on the geographic coordinates of each contaminant site, overlaying with residential address at birth. This study found that residing within 1 mi of a TRI facility that released solvents had a significantly elevated risk for CNS defects with an odds ratio (OR) of 1.3.

Neumann et al. (1998) attempted to create a method for incorporating the toxicity factors so that the TRI data are more useful in estimating concentrations in the environment and potential effects from exposure. The chronic toxicity index was developed by the U.S. EPA’s Region III Air Radiation and Toxics Division using the TRI databases and chronic oral toxicity factors and total mass for both carcinogens and noncarcinogens to estimate the relative hazards of TRI chemicals. The investigators used oral reference doses and cancer potency factors for the chronic toxicity index and ranked TRI chemicals on the basis of total mass versus total chronic toxicity index. The results varied greatly (Neumann et al. 1998). Even though the chronic toxicity index has its own limitations, it is likely to be a better indicator of potential risk than the use of mass alone.

The primary objective of this study was to investigate whether mothers of childhood brain cancer cases had greater potential residential exposure to TRI chemicals than control mothers during pregnancy. We assessed potential exposure by considering residential proximity to TRI facility during pregnancy, whether carcinogens were emitted, and a comparative ranking system for TRI chemical releases by combining toxicity information and total mass of release.

## Materials and Methods

### Study population

Subjects who participated in the U.S. Atlantic Coast childhood brain cancer study, a population-based case–control study of environmental risk factors (Agency for Toxic Substances and Disease Registry 2004), were eligible for the TRI study. Briefly, cases eligible for the original Atlantic coast childhood brain cancer study included all incident cases of first primary brain cancer [*International Classification of Diseases for Oncology* (ICD-O-2) (World Health Organization 1990) codes C71.0–C71.9 including all morphologic codes with a behavior code of 3, excluding lymphomas] (Percy et al. 1990) diagnosed at < 10 years of age between 1993 and 1997, born in the United States, and a resident of one of the four states (Florida, New Jersey, New York excluding New York City, and Pennsylvania) at the time of diagnosis. In addition, an eligible case had to have the biological mother available for an interview in English and a telephone in the household. During the computer-assisted telephone interview, a standardized screening questionnaire was used to verify eligibility and obtain mothers’ consent to participate in the study. The study protocol was approved by the Centers for Disease Control and Prevention and four state institutional review boards. The four statewide cancer registries initially identified 937 case children. Eligibility screening interviews were not completed for 228: three (0.3%) physician refusals, two (0.2%) out-of-state children, 176 (18.8%) unable to be traced, 39 (4.2%) mother refusals, and eight (0.9%) with language barriers. Of the 709 case children for whom screening interviews were completed, 662 met the eligibility criteria, and 535 mothers of the 662 agreed to participate. Of the 535, nine were excluded because of difficulties in finding matched controls, and 526 were included in the original study (56.1% of the originally identified 937 cases or 79.5% of the 662 eligible case children).

Potential controls in the original study were identified from the study base population through random-digit dialing (RDD) (Wacholder et al. 1992; Waksberg 1978). Eligible controls had to be born in the United States, be free of cancer, have the biological mother available for an interview in English, and have a telephone in the household. An equal number of controls were selected by matching individually to cases on sex, race (white, black, or other), birth year (± 1 year), and state of residence at the time of cases’ diagnosis. The age at diagnosis of each case was used as a reference age for the corresponding control. Among the 20,802 RDD numbers prescreened for nonworking and nonvoice numbers, each of 3,553 (17.1%) households had a child meeting the eligibility criteria for the control selection. Of the 3,553 children, 820 (23.1%) met the matching criteria. Of the 820 meeting the matching criteria, 122 did not have a matching case available, 102 mothers refused to participate, and 526 agreed to participate (2.5% of the 20,802 working residential numbers or 83.8% of the 628 eligible control children for whom a matching case child was available).

This TRI study included 764 subjects (382 case–control pairs) of the 1,052 (526 case–control pairs) participants in the original study: 222 subjects born before 1988 were excluded because the reporting of TRI information began in 1987; 34 subjects who had incomplete pregnancy residential information or dates of residence were excluded; 32 subjects missing their matched case or control counterparts were excluded.

### Computer-assisted telephone interview

The biological mothers of cases and controls were interviewed in English using a computer-assisted telephone interview system. Bilingual (Spanish) interviewers were available. Mothers were asked to provide information on residential history of the parents and child from 24 months before the child’s birth until the age of diagnosis or reference age (i.e., age at diagnosis for counterpart case) for controls. Interviewers were instructed to obtain residential addresses and to take nearest intersecting street names when the street numbers were unavailable. The questionnaire also included information on demographic characteristics and on mothers’ smoking habits during pregnancy.

### Exposure assessment

Addresses of mothers during pregnancy for the 10 months before birth were geocoded with latitude and longitude coordinates using GeoCoder (version 3.4b; GeoAccess Inc., Lenexa, KS). This software package was used with the TRI facilities’ geographic coordinates to determine exact distances from each residence to all facilities within a 2-mi radius. We geocoded 624 of 928 (67.2%) pregnancy addresses in the first round. We located 288 of 928 (31%) unable to be geocoded in the first round because of invalid addresses or zip codes using database records, public records, court records, and calls to post offices; these were gecoded in the second or third round. A total of 912 of 928 (98.3%) pregnancy addresses were successfully geocoded.

We extracted the TRI data from the U.S. EPA TRI CD-ROM containing information for the years 1987–1997 (U.S. EPA 2000). To assess the quality of geocoded data obtained from the TRI database, we randomly selected approximately 5% of the TRI facilities’ addresses used in this study and matched those to addresses on the Streetmap 2000 street layer residing on the spatial data engine using ArcGIS (version 8.1; Environmental Systems Research Institute, Inc., Redlands, WA). Distance measurements for both were calculated. The range of difference was between 0.017 and 0.534 mi, with a mean value of 0.3455 mi (0.060, 0.119, 0.343 mi for 25th, 50th, and 75th percentiles respectively). The original plan to use the 0.5-mi radius or cutoff point as a potential exposure category was abandoned because these distances were deemed unstable. We retained the 1.0-mi and 2.0-mi radii as proximity measures.

We calculated the distance from the mother’s residence during any point in pregnancy to the nearest TRI facility and categorized the exposure levels as residing ≤ 1 mi versus > 1 mi, and ≤ 2 mi versus residing > 2 mi of any facility. Next, we investigated whether any carcinogen was released to the air from facilities within 1 mi versus > 1 mi and within 2 mi versus > 2 mi of any facility. The TRI air emissions of any class of carcinogens were categorized as dichotomous variables without regard to the amount released to the air. Air emissions included stack and fugitive air releases. Carcinogens as defined by the U.S. EPA included all known, probable, and possible human carcinogens (U.S. EPA 2002a); EPCRA section 313 lists toxic chemicals that meet the Occupational Safety and Health Administration carcinogen standard and are associated with the 0.1% *de minimis* concentration limit when in a mixture (U.S. EPA 2002a).

Finally, we chose a hazard-screening tool for exposure assessment. To comparatively rank TRI chemical releases, we adapted the chronic toxicity index developed by the U.S. EPA’s Region III (Neumann et al. 1998). The screening tool uses the TRI databases combining toxicity factors and total mass to estimate the relative hazards of TRI chemical releases with a separate algorithm for carcinogens and noncarcinogens. For carcinogens, the carcinogenic weight of evidence (WOE) and cancer potency factors (CPF) and the pounds of chemicals released are included in the index calculation. The WOE data were obtained from the Integrated Risk Information System (U.S. EPA 2002b), a database of human health effects that may result from exposure to environmental substances. We used the U.S. EPA Region II Risk-Based-Concentration Table (U.S. EPA 2002a) to obtain the CPF for the inhalation or ingestion routes of exposure. Although the likely exposure route would be through the inhalation route, chemicals with only oral CPF were included in the index using the oral CPF value.

We modified the chronic toxicity index to include the duration of residence and the distance to the TRI facility. With some subjects’ pregnancy period spanning 2 calendar years, duration of residence at each address during pregnancy for each calendar year was calculated separately to match it with the appropriate year-specific TRI data. Because the TRI data report the total amount of emissions during a calendar year, the number of months a woman lived at a particular address for the particular year while pregnant was divided by 12 months and then multiplied into the chronic toxicity index. Only known, probable, and possible carcinogens, as defined by the U.S. EPA (2002a), that were released within 2 mi of pregnancy residence and having the appropriate carcinogenic WOE and CPF information available were included. We incorporated the duration of exposure, and residential distance to the facilities to the chronic toxicity index:


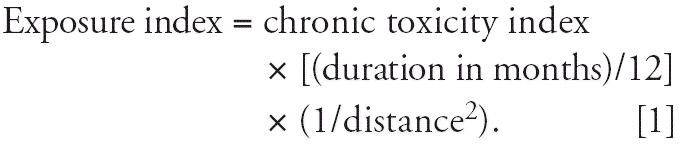


### Statistical analysis

We used conditional logistic regression analyses to achieve maximum likelihood estimates of ORs and 95% confidence intervals (CI) for the exposure variables. Exposure variables for residential proximity and residing near a facility releasing carcinogens were categorized as ≤ 1 mi versus > 1 mi, and ≤ 2 mi versus > 2 mi. We categorized the exposure index into three levels using the following cut-point values: zero; greater than zero but less than median index value among controls; and greater than median index value among controls. The potential confounders examined included mother’s education, household income level, and mother’s pregnancy age. Because there were no substantial confounding effects from these variables, judged by the change-inestimate methods (i.e., 10% change in OR), unadjusted ORs are presented. Because it is possible that the effect of potential gestational exposure may be more relevant to cancer development in earlier childhood or to particular histological subtype of childhood brain cancer, we repeated the analysis by reference age (< 5 and ≥ 5 years) and by two major histological subtypes, primitive neuroectodermal tumors (PNET) and astrocytomas [ICD-O-2 codes 9400–9441 and 9470–9473, respectively (Percy et al. 1990)]. All statistical analyses were conducted using SAS software (version 8.02; SAS Institute Inc., Cary, NC).

## Results

### Demographics and histopathologic characteristics of the study population

Table 1 shows the distribution of the histopathologic types of the brain tumor among cases and controls. Most of the case and control children were white (88%); 11% were black and only 1.6% were classified as other. There were 233 pairs (61%) with a reference age (age at diagnosis for cases) of < 5 years. Most of the children (72%) were born before 1993. The distribution of mothers’ age at pregnancy was similar in cases and controls (Table 1). Case mothers’ education levels were slightly higher than control mothers’ education levels, but the household income levels were slightly higher for the controls. Astrocytomas were the most common type; about half the cases had astrocytomas whereas 29% had PNETs (Table 2).

Overall, 635 case and control mothers lived at one address for the entire pregnancy. The remaining 129 (17%) mothers had lived at more than one address during pregnancy: 121 mothers with two and eight with three addresses. The resulting total was 901 addresses. Mothers’ residences during pregnancy were located in 23 different states for the case mothers and 18 different states for control mothers. However, 94% of both case and control mothers lived during the entire pregnancy in one of the four states—Florida, New Jersey, New York (excluding New York City), or Pennsylvania.

### Residential proximity to TRI facilities during pregnancy

We identified a total of 1,624 different TRI facilities within 2 mi of any of the case and control residences. The case mothers had a higher frequency of living within 1 and 2 mi of any TRI facility than control mothers at any point during pregnancy. Table 3 shows the results of analyses comparing cases and controls living within 1 mi versus > 1 mi from TRI facilities and living within 2 mi versus > 2 mi. Living within 1 mi of any TRI facilities during pregnancy showed slightly increased OR for all reference ages (OR 1.32; 95% CI, 0.96–1.80) and a statistically significant OR for those < 5 years of age at diagnosis (OR 1.66; 95% CI, 1.11–2.48) compared to living > 1 mi. For living within 1 mi versus > 1 mi from a TRI facility releasing carcinogens, the OR was 1.48 (95% CI, 1.01–2.17) for all ages, and 1.72 (95% CI, 1.05–2.82) for those < 5 years of age at diagnosis. Analysis by tumor types, astrocytoma and PNET, was associated with increased risk estimates, but the results were not statistically significant (Table 4). For astroycytoma, living within 1 mi of any TRI facility had an OR of 1.18 (95% CI, 0.77–1.82) compared to living > 1 mi from any facility, and living within 1 mi of a facility releasing carcinogens had an OR of 1.32 (95% CI, 0.79–2.22) compared to living > 1 mi from a facility releasing carcinogens.

### Exposure index

Of 193 TRI compounds classified as known, probable, or possible carcinogens, 55 compounds were actually released within 2 mi of residences of the study population during pregnancy. From those 55 compounds, we obtained information on 26 compounds and calculated the exposure indices for them. The most common compounds released within 2 mi of residence for individuals in the study population were dichloromethane, nickel and nickel compounds, styrene, lead, trichloroethylene (TCE), formaldehyde, and di(2-ethylhexyl) phthalate. Compounds with the highest exposure index values for residential addresses were 1,3-butadiene, ethylene oxide, dichloromethane, chloroform, and vinyl chloride.

There was an increasing risk trend as the exposure index level increased for those with a reference age of < 5 years: Compared to subjects with an exposure index of zero, the ORs were 1.24 (95% CI, 0.67–2.28) for subjects with an exposure index of greater than zero and less than the median and 1.25 (95% CI, 0.67–2.34) for subjects with an exposure index of greater than the median (Table 5). However, the increasing trend was not statistically significant (*p* = 0.38). No increasing trend in risks for two major subtypes of brain cancer, astrocytoma and PNETs, was observed by the increasing exposure index level (Table 6).

Because some of the carcinogens did not have the appropriate toxicity information, a separate analysis was conducted by calculating the exposure index only with the mass of compounds released, duration at each residence, and distance to the facility. However, the results did not differ and elevated risk was not observed.

## Discussion

Environmental epidemiology studies constantly struggle with ways to assess past exposure. Although a number of databases include information on the release of chemicals, these were collected mostly for regulatory purposes and therefore lack the individual specificity desired for these studies. Nonetheless, it is important to try to use these data in creative ways if we are to have any information at all on past exposures. Because of the uncertainty built into using these data, studies such as this must be interpreted with caution. In this study we used data from the TRI to assess exposure in three different ways: living within a specified distance of a TRI facility (1 or 2 mi), living within a specified distance of a TRI facility emitting a carcinogen (1 or 2 mi), and a toxicity index that took into consideration the toxicity of the chemical released and the duration of the exposure in addition to distance from a TRI facility. Actual individual exposure measures for specific chemicals were not available for this study.

We observed an elevated risk for mothers living within 1 mi of a TRI facility and living within 1 mi of a facility releasing carcinogens for having children with brain cancer diagnosed before 10 years of age. The odds ratios were higher for brain cancer cases diagnosed before age 5 years. For the exposure assessment using the exposure index, we observed an increasing risk trend as the exposure index level increased, although the trend was not statistically significant. Nevertheless, since the number of subjects that actually had a positive exposure index value was small, *p*-values would have been affected by the small sample size.

It is not feasible to compare the results of this current analysis with previous studies because similar studies linking childhood brain cancers with TRI releases are not available. However, similar methods of exposure assessment were used in previous studies on central nervous system birth defects from possible exposure to TRI sites (Croen et al. 1997; Marshall et al. 1997). Marshall et al. (1997) observed an increased risk for CNS defects associated with living within 1 mi of a facility emitting either solvents or metals into the air; however, they did not observe a dose–response trend as distance to TRI facilities was reduced. It is interesting that the 1-mi cutoff for exposure categorization resulted in significant risk for CNS defects (Marshall et al. 1997), but there was a lack of association when distance was further subcategorized within 1 mi.

Although prenatal residential proximity to TRI facilities resulted in a statistically significant increased risk for childhood brain cancer, it is imprudent to associate that with actual exposure to any compounds released, so results should be interpreted accordingly. Several issues concerning exposure assessment must be taken into account. Some of the limitations of this analysis include concern over accuracy of residential history data, limitations of the TRI data themselves, and methods of exposure assessment.

Residential history information used in this analysis comprised self-reported responses from mothers of cases and controls. There is potential for recall and reporting bias that is further compounded by the fact that some subjects had to provide information dating back 10 years. Inaccurate address information for cases and controls that made it impossible to assign geocoding information meant that distances to TRI facilities could not be determined, so that some cases and controls had to be excluded from the study. The concern here is selection bias, because subjects who were living in rural areas, less educated, or frequent movers may have been more likely to be excluded (Ward et al. 2000). However, only 11 of the 830 children born after 1988 were missing information on distance to TRI facilities, and 23 of the 830 children were missing mothers’ pregnancy residential information, for a total of only 34 of the 830 (4%), which is likely too small of a number to introduce such a bias.

Another limitation of this study lies with the TRI data themsleves: They are self-reports from companies and it is difficult to assess the accuracy of the data. Facilities with < 10 full-time employees or those not meeting TRI quantity thresholds are not required to report releases. Thus exposure experienced by both cases and controls may be higher than estimated through the TRI, because such facilities also may contribute to the overall pollutant burden in the community. The variability in exposure arising from these unreported emissions relative to those arising from TRI facilities is unknown. Also, chemical releases and waste generation are estimated and do not provide measurement of actual concentrations in the environment (Neumann et al. 1998).

For the first two levels of analysis using proximity and the release of carcinogens, residing near multiple facilities or multiple compounds released was not accounted for, although an attempt was made to include them in the exposure index. The exposure index has its own limitations because not all the TRI compounds have a toxicity value necessary for obtaining the chronic index. Several compounds lacked the inhalation data requiring oral toxicity factors to be used to estimate the index. However, preliminary findings suggest that substituting oral factors for inhalation did not change the final rank of TRI emission using the chronic index approach (Neumann et al. 1998).

This study did not account for other potential confounders such as mother’s exposure to chemicals at the workplace during pregnancy. The TRI is just one source of information on environmental releases. Other sources of air pollution such as toxic emissions from cars or other hazardous waste sites were not included. Only TRI air emissions data were extracted for the analysis, so we did not explore possible exposure through contaminated drinking water. The pathway of exposure through contaminated drinking water is more difficult to assess for each individual; the location of TRI sites may or may not have resulted in water contamination because municipal water wells are not directly related to location of residences (Marshall et al. 1993). We would need to know whether private wells or municipal water wells were the principal source of water and determine if they were possibly contaminated by TRI chemical releases.

Although we used only the period of 10 months before birth, many mothers and their children lived in the same residential address long after birth but these exposure data were not included in the analysis. Therefore, it is difficult to rule out effects of potential exposure after birth. Further studies may be conducted to determine whether children who had lived at the same address from pregnancy to early childhood may have been exposed to further environmental chemical releases and possibly had a higher risk than those exposed only prenatally. Furthermore, because the TRI facilities report the annual releases and transfer without indicating the specific time and date of the release, it is possible that the actual releases occurred outside of the 10-month pregnancy period we examined.

There are several strengths in this study. This is the only study to date to examine the role of TRI releases and childhood brain cancer. In addition, this study included a large number of cases and controls drawn from the general population. We attempted to improve and build on previous exposure assessment methods. Most previous studies such as those dealing with environmental equity have compared populations using census tracts and circular zones of different distances around hazardous waste sites and compared population characteristics within and outside of those boundaries (Sheppard et al. 1999). Some have used ZIP-code boundaries (White and Aldrich 1999); however, ZIP codes have irregular boundaries, which do not indicate any specific relation to the hazardous waste site. We used direct distance to the TRI facilities and attempted to incorporate the amount as well as the toxicity of compounds released through the use of the chronic toxicity index.

Most published studies relied on the address on the birth certificate, which may not give a true picture of residence throughout the entire pregnancy. In this study we used residential addresses during pregnancy that were obtained from a survey question on residential history, rather than using the address at time of birth, and included multiple addresses when applicable.

Our results suggest a possible relationship between living within 1 mi of any TRI facility or a TRI facility emitting carcinogens during pregnancy and a child’s later developing childhood brain cancer. However, there are many uncertainties as to why such a relationship exists and why the same relationship was not found for living within half a mi of a facility. Most of the limitations discussed would be expected to bias the risk estimates toward the null and obscure any true association; however, it is unclear how other limitations might affect the risk estimate.

Despite the inherent limitations in using these data for epidemiology studies, research in this area needs to continue to refine their use. Further studies need to be conducted to explore whether these results can be replicated and also address and improve on some of the limitations described. Although this was a large study of childhood brain cancer including > 300 cases and 300 controls, this study was not designed to focus on specific chemicals because the number of cases and controls with potential to exposure specific carcinogens would be too small to warrant meaningful analysis. Therefore, it is not possible to pinpoint the specific agents that may have increased the risk for brain cancer. There is the potential for further improving on exposure assessment methods by using an exposure index using a larger sample size or by obtaining more complete toxicity and exposure information for the compounds.

## Correction

In Table 2, the value for “All other” has been corrected from 17 (4.5), as published online, to 75 (19.6); in Table 5, the value for “All reference ages” exposure index level II has been changed from 1.91 to 0.91.

## Figures and Tables

**Table 1:** Distribution of characteristics of the study population [no. (%)].

Characteristic	Cases	Controls
All	382 (100)	382 (100)
Sex		
Male	229 (60.0)	226 (59.2)
Female	153 (40.1)	156 (40.8)
Birth year		
1988–1992	275 (72.0)	275 (72.0)
1993–1997	107 (28.0)	107 (28.0)
Reference age^a^ (years)		
<5	233 (61.0)	233 (61.0)
5–9	149 (39.0)	149 (39.0)
Race		
White	335 (87.7)	335 (87.7)
Black	41 (10.7)	41 (10.7)
Other	6 (1.57)	6 (1.57)
Residence state at reference year		
Florida	113 (29.6)	113 (29.6)
New Jersey	90 (23.6)	90 (23.6)
New York	75 (19.6)	75 (19.6)
Pennsylvania	104 (27.2)	104 (27.2)
Mother’s education		
≤ High school	117 (30.6)	127 (33.2)
Some college	123 (32.2)	138 (36.1)
College graduate	89 (23.3)	76 (19.9)
Postcollege	52 (13.6)	41 (10.7)
Missing	1 (0.26)	
Household income (US$/year)		
20,000	57 (14.9)	46 (12.0)
20,000–50,000	123 (32.2)	117 (30.6)
> 50,000	171 (44.8)	193 (50.5)
Missing	31 (8.1)	26 (6.8)
Mother’s age at pregnancy (years)		
< 20	21 (5.5)	20 (5.2)
20–24	62 (16.2)	62 (16.2)
25–29	112 (29.3)	103 (27.0)
30–34	114 (29.8)	116 (30.4)
≥ 35	73 (19.1)	76 (19.9)
Missing		5 (1.3)

a Age at diagnosis for cases and matched age for controls.

**Table 2:** Central nervous system tumor types.

Morphology type	ICD-O-2 Codes	No. (%)
Astrocytoma	9400–9441	195 (51.0)
Primitive neuroectodermal tumors	9470–9473	112 (29.3)
All other		75 (19.6)
Total		382

**Table 3:** ORs for residential proximity to any TRI facility and facility releasing carcinogen(s) during pregnancy for childhood brain cancer by reference age.

	All reference ages		Reference age < 5 years	
	Ratio of discordant pairs	OR (95% CI)	Ratio of discordant pairs	OR (95% CI)
Proximity to any TRI facility^a^				
≤ 1.0 mi vs. > 1.0 mi	91/69	1.32 (0.96–1.80)	63/38	1.66 (1.11– 2.48)
≤ 2.0 mi vs. > 2.0 mi	86/75	1.15 (0.84–1.56)	51/45	1.13 (0.76–1.69)
Proximity to TRI facility releasing carcinogen(s)^b^				
≤ 1.0 mi vs. > 1.0 mi	65/44	1.48 (1.01–2.17)	43/25	1.72 (1.05–2.82)
≤ 2.0 mi vs. > 2.0 mi	89/82	1.09 (0.80–1.47)	51/50	1.02 (0.69–1.51)

a Lived within the set distance of any TRI facility(s) at any point during pregnancy.

b Any air releases of known, probable, and possible carcinogens as defined by the U.S. EPA (2002a).

**Table 4:** ORs for residential proximity to any TRI facility and facility releasing carcinogen(s) during pregnancy for childhood brain cancer by histological types.

	Astrocytoma		Primitive neuroectodermal tumors	
	Ratio of discordant pairs	OR (95% CI)	Ratio of discordant pairs	OR (95% CI)
Proximity to any TRI facility^a^				
≤ 1.0 mi vs. > 1.0 mi	45/38	1.18 (0.77–1.82)	25/18	1.39 (0.76–2.55)
≤ 2.0 mi vs. > 2.0 mi	45/37	1.22 (0.79–1.88)	21/29	0.72 (0.41–1.27)
Proximity to TRI facility releasing carcinogen(s)^b^				
≤ 1.0 mi vs. > 1.0 mi	33/25	1.32 (0.79–2.22)	15/14	1.07 (0.52–2.22)
≤ 2.0 mi vs. > 2.0 mi	41/40	1.03 (0.66–1.59)	24/32	0.75 (0.44–1.27)

a Lived within the set distance of any TRI facility(s) at any point during pregnancy.

b Any air releases of known, probable, and possible carcinogens as defined by the U.S. EPA (2002a).

**Table 5:** ORs (95% CIs) for the exposure index categories for childhood brain cancer by reference age.

Exposure index level^a^	All reference ages	Reference age < 5 years
I	1.00	1.00
II	0.91 (0.56–1.46)	1.24 (0.67–2.28)
III	1.33 (0.85–2.09)	1.25 (0.67–2.34)
Trend test	*p* < 0.25	*p* < 0.38

Level I: subjects with an exposure index of zero; level II: subjects with an exposure index of > 0 and < 50% percentile; level III: subjects with an exposure index of > 50% percentile.

a For selected carcinogens released within 2 mi of residence: (chronic index × duration of residence) × (1/distance^2^).

**Table 6:** ORs (95% CIs) for the exposure index categories for childhood brain cancer by histological types.

Exposure index level^a^	Astrocytoma	Primitive neuroectodermal tumors
I	1.00	1.00
II	0.70 (0.33–1.50)	0.40 (0.16–1.03)
III	1.23 (0.66–2.27)	1.05 (0.46–2.39)
Trend test	*p* < 0.65	*p* < 0.50

Level I: subjects with an exposure index of zero; level II: subjects with an exposure index of > 0 and < 50% percentile; level III: subjects with an exposure index of > 50% percentile.

a For selected carcinogens released within 2 mi of residence: (Chronic index × duration of residence) × (1/distance^2^).
